# Analgesic, anti-inflammatory and anti-ulcer properties of Thai *Perilla frutescence* fruit oil in animals

**DOI:** 10.1042/BSR20203166

**Published:** 2021-01-22

**Authors:** Narisara Paradee, Pimpisid Koonyosying, Winthana Kusirisin, Rattanaporn Janthip, Duangta Kanjanapothi, Kovit Pattanapanyasat, Somdet Srichairatanakool

**Affiliations:** 1 Oxidative Stress Cluster, Department of Biochemistry, Faculty of Medicine, Chiang Mai University, Chiang Mai, Thailand; 2 Department of Family Medicine, Faculty of Medicine, Chiang Mai University, Chiang Mai, Thailand; 3 Faculty of Pharmacy, Payap University, Chiang Mai, Thailand; 4 Department of Research and Development, Faculty of Medicine, Siriraj Hospital, Mahidol University, Bangkok, Thailand

**Keywords:** analgesic, anti-inflammatory, anti-ulcer, fruit oil, Perilla frutescence

## Abstract

*Perilla frutescens* fruit oil (PFO) is rich in α-linolenic acid (ALA) and exhibits biological activities. We aimed to investigate analgesic, anti-inflammatory and anti-ulcer activities of PFO and PFO-supplemented soybean milk (PFO-SM) in animal models. Analgesic activity was assessed in acetic acid-induced writhing in mice, while anti-inflammatory activity was performed in ethyl phenylpropiolate (EPP)-induced ear edema and carrageenan-induced hind paw edema in rats. Anti-ulcer effects were conducted in water immersion stress, HCl/ethanol and indomethacin-induced gastric ulcer in rats. Distinctly, PFO, containing 6.96 mg ALA and 2.61 mg LA equivalence/g, did not induce acute toxicity (LD_50_ > 10 mL/kg) in mice. PFO (2.5 and 5 mL/kg) and PFO-SM (0.05 mL PFO equivalence/kg) inhibited incidences of writhing (16.8, 18.0 and 32.3%, respectively) in acetic acid-induced mice. In addition, topical applications of PFO (0.1 and 1 mL/ear) significantly inhibited EPP-induced ear edema (59.3 and 65.7%, respectively) in rats, while PFO-SM slightly inhibited ear edema (25.9%). However, PFO and PFO-SM did not inhibit carrageenan-induced hind paw edema in rats. Indeed, PFO (2.5 and 5 mL/kg) significantly inhibited gastric ulcers in rats that induced by water immersion stress (92.4 and 96.6%, respectively), HCl/ethanol (74.8 and 73.3%, respectively) and indomethacin (68.8 and 88.9%, respectively), while PFO-SM did not. PFO displayed potent analgesic, anti-inflammatory and anti-ulcer properties, while PFO-SM exerted only analgesic properties. Thus, Thai PFO and its functional drink offer potential benefits in treatment of analgesic, inflammatory diseases and gastric ulcer.

## Introduction

Inflammation, which can be caused by physical and chemical trauma, drugs and infectious microorganisms, is a defense mechanism of the body that serves to eliminate damaged tissue or invading pathogens [1]. Commonly, related responses are characterized by localized redness, heat, swelling, pain and a loss of tissue function. During these responses, vascular permeability is increased to recruit inflammatory cells to the injury sites, which can cause leakage of plasma fluid and subsequent edema. In addition, released inflammatory mediators, such as histamine and prostaglandins (PGs), can promote the occurrence of pain. Finally, edema and pain can cause a loss of tissue function. However, uncontrolled inflammation contributes to a variety of inflammatory diseases including cardiovascular disease, diabetes and gastric ulcers [2,3].

Currently, gastric ulcers have become a serious health problem throughout the world. Gastric ulcers occur in the mucosa of stomach as a result of inflammation and oxidative stress [4,5]. Normally, mucus and bicarbonates that serve as a protective layer, are secreted by mucosal cells to neutralize hydrochloric acid (HCl); nonetheless, this layer can be broken down by the actions of non-steroidal anti-inflammatory drugs (NSAIDs), *Helicobacter pylori* and ethanol [6–8]. Moreover, an increase in stomach acid secretions also promotes gastric ulcers that can be caused by stress, smoking and the consumption of spicy foods [5]. Deep ulcers can damage underlying blood vessels leading to bleeding and hemorrhaging in the gastrointestinal tract [7]. Thus, treatment of gastric ulcers should focus on the restoration of this protective layer and decrease the presence of gastric acid in order to minimize tissue damage.

*Perilla frutescens* (L.) Britton are grown in Northern provinces of Thailand including Chiang Mai, Maehongsorn and Nan (Thai local name ‘nga‐keemon’). Perilla fruits have been widely used in both traditional and medicinal foods. Perilla fruit oil (PFO) has been used as Northern food additive and herb in folk medicine for a long time. Nowadays, PFO is formulated in dessert, capsule gel, functional drink and soap for commercial purposes. Interestingly, the fruits contain approximately 35–45% oil that is rich in ω3 polyunsaturated fatty acid (ω3-PUFA), particularly α-linolenic acid (ALA) (54–65% of total fatty acids) [9–11]. In addition, ω6-PUFA, namely linoleic acid (LA), and ω9 PUFA, namely oleic acid (OA), have been detected in PFO [10]. Likewise, PFO contains certain other lipids including glycolipids, phospholipids, sterols and tocopherols [10,12]. Evidences from animal studies suggest that PFO possesses anti-asthmatic and neuroprotective effects [9,13,14]. Indeed, PFO modulated lipid parameters and pro-inflammatory cytokines, potentially lowering incidences of cardiovascular disease and inflammatory disease [15–17]. We have recently demonstrated that Thai PFO showed hepatoprotective effects against carbon tetrachloride (CCl_4_)-induced liver inflammation in rats [18].

The dietary beverage, soybean milk (SM), is a popular traditional drink that is rich in protein, essential amino acids, isoflavones (e.g. daidzein, glycitein, genistein, daidzin, glycitin and genistin) and lecithin [19,20]. SM has been found to exert analgesic, anti-inflammatory and cardiovascular effects [21–23]. Therefore, a combination of PFO and SM would promote a range of nutritional and pharmacological effects on human health. Many previous studies have demonstrated the biological activities of PFO; nonetheless, only a few studies have been published on the analgesic, anti-inflammatory and anti-ulcer effects of PFO and PFO-supplemented SM (PFO-SM). In the present study, PFO and PFO-SM were evaluated in terms of its analgesic, anti-inflammatory and anti-ulcer activities in animals.

## Materials and methods

### Chemicals and reagents

All organic solvents (the highest pure grade) were purchased from Fisher Scientific Company (Hampton, NH, U.S.A.). Acetic acid and HCl were purchased from Merck KGaA Company (Darmstadt, Germany). Acetylsalicylic acid (ASA), 4-bromomethyl-7-methoxycoumarin (Br-MMC), carrageenan, cimetidine, 1,4,7,10,13,16-hexaoxacyclooctadecane (18-crown-6), ethyl phenylpropiolate (EPP), dimethylsulfoxide (DMSO), phenylbutazone (PBZ) and indomethacin were obtained from Sigma–Aldrich Chemicals Company (St Louis, MO, U.S.A.). Pentobarbital sodium was prescribed by a pharmacist and obtained from a local drug store in Maharaj Nakorn Chiang Mai Hospital, Faculty of Medicine, Chiang Mai University, Chiang Mai, Thailand. Palsgaard® RecMilk 121 (Palsgaard A/S Company, Juelsminde, Denmark), sodium chloride (Chemiphan Corporation Company, Limited, Bangkok, Thailand) and natural cane sugar (Mitrphol Sugar Group, Limited, Bangkok, Thailand) (Food Grade) were purchased from Tesco Supermarket in Chiang Mai, Thailand.

### Preparation of PFO and PFO-SM

Perilla fruits were harvested from fields located in Wienghang District, Chiang Mai, Thailand (botanically examined by Dr. C. Leon, Royal Botanic Gardens Kew, and compared botanically and chemically with authentic *P. frutescens* fruits from the Royal Botanic Gardens Kew Economic Botany Collections, voucher reference: EBC 81840 TCMK 411) and underwent regular pressing in order to yield PFO (27%, w/w yield). Yellow soybeans (*Glycine max* L.), which cultivated in Doi Saked District, Chiang Mai, were bought from a grocery. Each batch of SM was prepared according to the method established by Jiang et al. [24] with slight modification. Briefly, dry soybeans were rinsed and soaked in clean tap water (dry beans/water = 1/5, w/v) for 24 h at room temperature. The mixture was then subjected to a household soy milk maker (Model FDM-Z100, Tojay Food Machinery Company (Limited), Henan, People Republic of China) to obtain aqueous SM extract at 60–70°C. The resulting soybean slurry was filtered through a mesh screen to obtain SM, then brought to a boil at 80°C for a period of 15–20 min to improve its nutritional value and flavor and to inactivate the trypsin inhibitor. The cooked product was immediately placed in an ice bath to allow it to cool down rapidly and enough water was added to achieve the desired solids content of the final product. PFO-SM was prepared with the most suitable compositions by homogenously mixing SM (85%, v/v), cane sugar (5%, w/v), PFO (1%, v/v), sodium chloride (0.1%, w/v) and a stabilizer/emulsifier (0.125% Palsgaard® RecMilk 121, w/v). The PFO was then subjected to fatty acid analysis, an acute toxicity study and pharmacological tests, while PFO-SM was subjected to pharmacological tests.

### High-performance liquid chromatography (HPLC) analysis of ALA and LA contents

ALA and LA were quantified using the method established by Prommaban et al. [25]. Briefly, PFO and authentic fatty acids, including ALA and LA, were reconstituted in acetonitrile (final volume 100 µL) and incubated with a derivatizing reagent (200 µL) made up of 10 mg Br-MMC, 26.5 mg 18-crown-6 and 100 mg potassium carbonate in 10 mL of acetonitrile at 60°C for 15 min in order to produce a fluorogenic methyl-7-methoxycoumarin fatty acid (MMC-FA) derivative. The derivative solution was passed through a 0.45-μm nylon-membrane filter and analyzed using the high-performance liquid chromatography/fluorescence detection (HPLC/FLD) system. The conditions included a column (C18 type, 4.6 mm × 250 mm, 5-μm particle size, Agilent Technologies, Santa Clara, California, United States), a mobile-phase solvent (acetonitrile:DI = 85:15, v/v), a flow rate of 1.5 mL/min, fluorescence detection (λ_ex_ 325 nm, λ_em_ 398 nm) and a data recorder/integrator using Millenium 32 HPLC Software. ALA and LA in PFO were identified by comparison with the specific retention time (T_R_) of the authentic standard. The resulting concentrations were then determined by the standard curves constructed from different concentrations.

### Animal care

Swiss albino mice and Sprague–Dawley rats were purchased from the National Laboratory Animal Center, Mahidol University (Salaya Campus), Bangkok, Thailand. The animals were housed in individual cages and were given free access to water and fed a commercial diet (No. C.P. 082, Perfect Companion Group Co. Ltd., Thailand). The animals were maintained at room temperatures in a range of 20–22°C with 50 ± 10% humidity and a 12-h light/dark cycle. The animals were acclimatized to laboratory conditions for 1 week prior to being subjected to the experiments.

### Acute oral toxicity study

Acute oral toxicity assessment of PFO in mice was performed according to the Organization of Economic Cooperation and Development (OECD) guideline 423, 2001 by the Institute of Medicinal Plant Research (Certified ISO/IEC 17025: 2005), Department of Medical Science, Ministry of Public Health, Nonthaburi, Thailand. In this procedure, female mice (40–60 g) were fasted for 3–4 h and weighed prior to dosing. PFO was orally administered in a single dose of 10 mL/kg to mice (*n*=3) with a gavage needle. After administration of PFO, the mice were observed individually for mortality, changes in behavior and physical appearance, as well as for signs of toxicity during the first 24 h with special attention being given to the first 4 h and daily thereafter for a total of 14 days. At the end of the experiments, all mice were killed by cervical dislocation and subject to gross necropsy. Internal organs were excised and were then macroscopically examined.

### Evaluation of analgesic activity in mice

Acetic acid-induced writhing in mice was initiated using the previously established protocols [26,27]. Male mice (40–60 g) were randomly divided into five groups (*n*=6 each) and were orally given deionized water (DI, 5 mL/kg), acetylsalicylic acid (ASA, 150 mg/kg), PFO (2.5 and 5 mL/kg) and PFO-SM (0.05 mL PFO equivalence/kg). After 30 min, all mice were intraperitoneally injected with 0.75% (v/v) acetic acid (10 mL/kg) to induce writhing. Writhing was indicated by stretching of the abdomen, extension of the hindlimbs or turning of the trunk (twisting). Incidences of writhing were counted for 15 min after an injection of acetic acid was administered for 5 min. Percentage of inhibition of the incidences of writhing was calculated.

### Evaluation of anti-inflammatory activity in rats

#### EPP-induced ear edema model

EPP is an irritating agent that induces a release of inflammatory and pro-inflammatory mediators, leading to an increase in vascular permeability and edema. EPP-induced ear edema in rats was conducted according to the method described by Brattsand et al. [28]. Male rats (60–80 g) were randomly divided into five groups (*n*=6 each). DMSO (5% in acetone, 1 mL/ear), phenylbutazone (PBZ, 1 mg/20 µL/ear), PFO (0.1 and 1 mL/ear) and PFO-SM (0.01 mL PFO equivalence/ear) were topically applied to the inner and outer surfaces of both ears. Ear edema was then induced by topical application of EPP (1 mg/20 µL/ear) and edema thickness was measured using a digital Vernier caliper before EPP application and at 15, 30, 60 and 120 min after EPP application. Percentage of inhibition of ear edema was then calculated.

#### Carrageenan-induced hind paw edema model

Carrageenan is a chemical that stimulates a release of inflammatory and pro-inflammatory mediators and is known to promote edema and an inflammatory response. Carrageenan-induced hind paw edema in rats was performed according to the method described by Winter et al. [29]. Male rats (100–120 g) were randomly divided into five groups (*n*=6 each) and fed orally with DI (5 mL/kg), ASA (300 mg/kg), PFO (2.5 and 5 mL/kg) and PFO-SM (0.05 mL PFO equivalence/kg). After 1 h, paw edema was induced by intradermal injection of 1% carrageenan (50 µL/paw) into the right hind paws of rats and the volume was measured using a plethysmometer before and at 1, 3 and 5 h after carrageenan injection. Percentage of inhibition of paw edema was then calculated.

### Evaluation of anti-ulcer activity in rats

#### Water immersion stress-induced gastric ulcer model

Water immersion stress-induced gastric ulcers in rats were initiated according to the previously described method [30]. Male rats (180–200 g) were randomly divided into five groups (*n*=6 each). All rats were fasted for 48 h prior to the experiment but were given free access to water except for 1 h before the experiment. Rats were fed orally with DI (5 mL/kg), cimetidine (100 mg/kg), PFO (2.5 and 5 mL/kg) and PFO-SM (0.05 mL PFO equivalence/kg) using a gavage needle. After 1 h, each rat was restrained individually in a cage and immersed up to its xiphoid in a temperature-controlled water bath (22°C) for a period of 5 h for induction of gastric ulcer. All rats were killed by intraperitoneal injection of pentobarbital sodium (40 mg/kg). Stomachs of the rats were excised, opened along the great curvature and rinsed with normal saline solution. The lengths of the gastric lesions were determined under light microscopy and expressed as the sum of the lesion length. Percentage of inhibition of the gastric ulcers was then calculated.

#### HCl/ethanol-induced gastric ulcer model

HCl/ethanol-induced gastric ulcer in rats was initiated according to the previously described method [8]. Male rats (180–200 g) were randomly divided into five groups (*n*=6 each). All rats were fasted for 48 h but were given free access to water except for 1 h before the experiment. Rats were then fed with DI (5 mL/kg), cimetidine (100 mg/kg), PFO (2.5 and 5 mL/kg) and PFO-SM (0.05 mL PFO equivalence/kg) using a gavage needle. After 1 h, all rats were orally administered with 36.5% HCl in ethanol (5 mL/kg) for induction of gastric ulcers over a period of 1 h. At the end of the study, all rats were killed by intraperitoneal injection of pentobarbital sodium (40 mg/kg). Their abdomens were opened and their stomachs were then excised and rinsed with normal saline solution. The lengths of gastric lesions were determined under light microscopy and expressed as the sum of lesion length. Percentage of inhibition of gastric ulcer was then also calculated.

#### Indomethacin-induced gastric ulcer model

Indomethacin-induced gastric ulcer in rats was performed according to the method established by Djahanguiri et al. [31]. Male rats (180–200 g) were randomly divided into five groups (*n*=6 each). All rats were fasted for 48 h but were given free access to water except for 1 h before the experiment. Rats were then treated with DI (5 mL/kg), cimetidine (100 mg/kg), PFO (2.5 and 5 mL/kg) and PFO-SM (0.05 mL PFO equivalence/kg) using a gavage needle. After 1 h, gastric ulcer was induced by a single subcutaneous injection of indomethacin (30 mg/kg) into the abdomen of rats. After 5 h, they were killed by intraperitoneal injection of pentobarbital sodium (40 mg/kg) and their stomachs were dissected. The lengths of gastric lesions were determined under light microscopy and expressed as the sum of lesion length. Percentage of inhibition of gastric ulcer was then calculated.

### Statistical analysis

Data are expressed as mean ± standard deviations (SD). Statistical significance was analyzed using one-way analysis of variance (ANOVA) with post-hoc Duncan, for which *P*<0.05 was considered significant.

## Results

### ALA and LA contents in PFO

**Figure 1:**
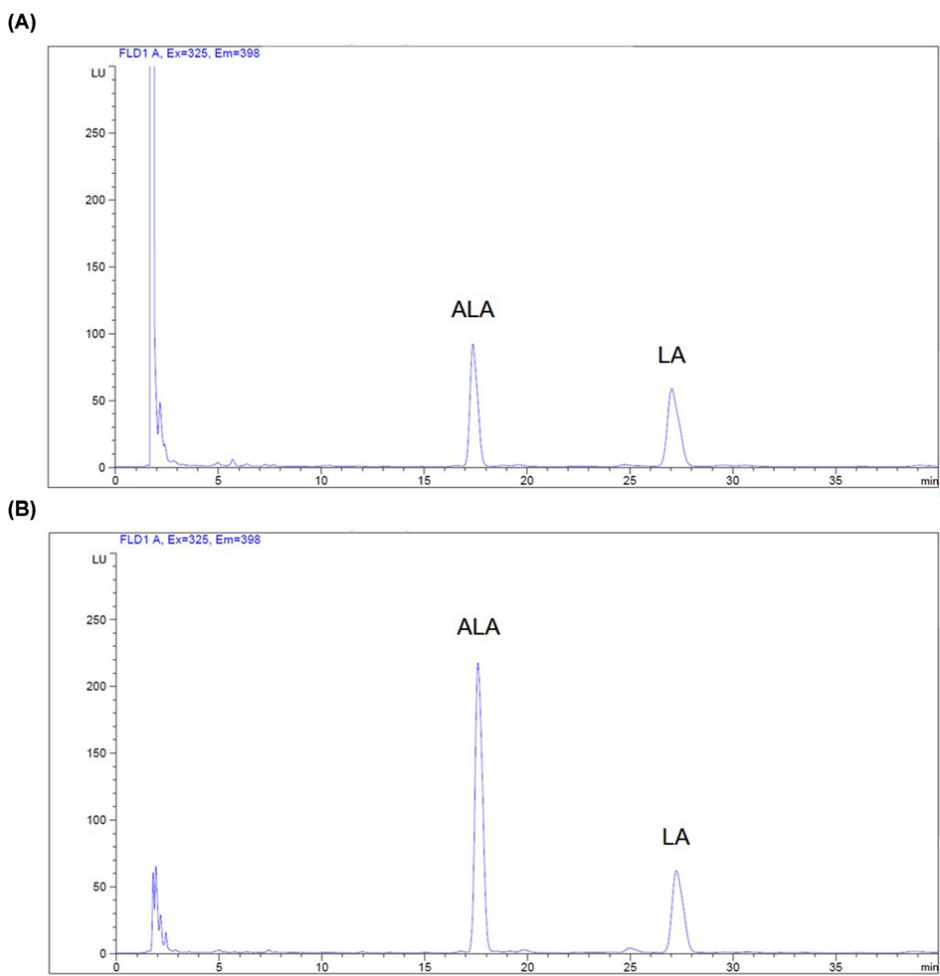
Fatty acid composition of PFO HPLC analysis of ALA and LA in authentic standards (**A**) and PFO (**B**). The samples were derivatized with the mixture of Br-MMC and 18-crown-6 to produce a fluorogenic methyl-7-methoxycoumarin fatty acid derivative, fractionated on the C18 type column, eluted with a mobile solvent (acetonitrile:DI), and detected the fluorescence. ALA and LA in PFO concentrations were determined from their standard curves.

ALA and LA in PFO were identified by comparison of their T_R_ values with those of the authentic standards, and the resulting concentrations were then determined using the calibration curves obtained from different concentrations. Consistent with the standard values, ALA and LA of PFO appeared at the T_R_ points of 17.59 and 27.24 min, respectively (Figure 1), for which their concentration values were 6.96 and 2.61 mg/g, respectively.

### Acute toxicity of PFO in mice

A single administration of PFO (10 mL/kg) did not cause mortality, changes in behavior or clinical signs of toxicity in mice during 14 days of observation. Gross necropsy findings did not show any abnormalities or histopathological changes in the internal organs. The results imply that the median lethal dose (LD_50_) of PFO was estimated to be more than 10 mL/kg.

### Analgesic effect of PFO and PFO-SM on acetic acid-induced writhing in mice

**Figure 2:**
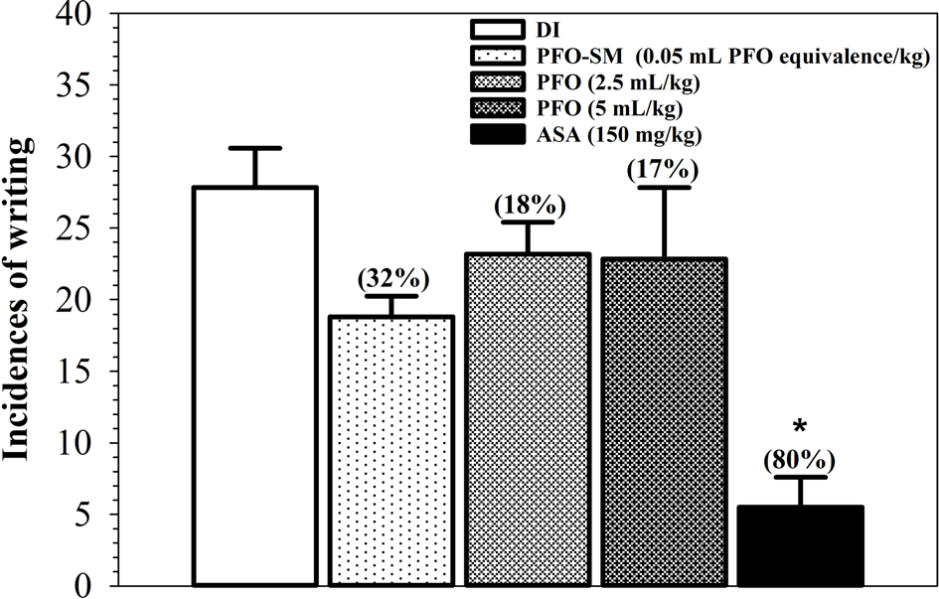
Analgesic effects of PFO, PFO-SM and ASA on acetic acid-induced writhing responses in mice Male mice (*n*=6 each) were orally given DI, PFO (2.5 and 5 mL/kg), PFO-SM (0.05 mL PFO equivalence/kg) and ASA (150 mg/kg) for 30 min, then intraperitoneally injected with acetic acid (0.75%, 10 mL/kg), and recorded incidences of writhing for 15 min. Statistics was performed by one-way ANOVA test, which *P*<0.05 was considered significance. Numbers of writhing are expressed as mean ± SD. *P<0.05 when compared with DI. Numbers in brackets represent percentage of inhibition of the writhing.

As shown in Figure 2, acetic acid induced approximately 28 incidences of writhing in mice. Pretreatment with ASA, which is a standard anti-inflammatory drug, significantly decreased the number of incidences of writhing and showed 80% inhibition of writhing when compared with the DI control group. Interestingly, PFO at doses of 2.5 and 5 mL/kg reduced the number of incidences of writhing (16.8 and 18% inhibition, respectively), while PFO-SM (0.05 mL PFO equivalence/kg) reduced the number of incidences of writhing by 32.3% inhibition. The results demonstrated that PFO and PFO-SM displayed analgesic activity through the inhibition of writhing in mice, in which PFO-SM was found to be more potent than PFO.

### Anti-inflammatory activity of PFO and PFO-SM

#### EPP-induced ear edema in rats

**Figure 3:**
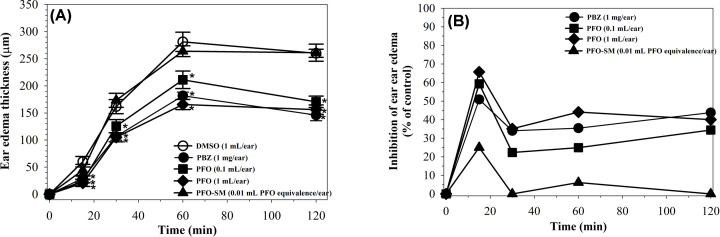
Anti-inflammatory effects of PFO, PFO-SM and PBZ on EPP-induced ear edema in rats DMSO, PBZ (1 mg/ear), PFO (0.1 and 1 mL/ear) and PFO-SM (0.01 mL PFO equivalence/ear) were topically applied to the inner and outer surfaces of rat’s ears, then induced by topical application of EPP (1 mg/20 µL/ear), and measured ear edema thickness before and after EPP application using a digital vernier caliper. Statistics was performed by one-way ANOVA test, which *P*<0.05 was considered significance. Data of edema thickness are expressed as mean ± SD, *P<0.05 when compared with DMSO (**A**). Percentage of inhibition of ear edema are shown (**B**).

Topical applications of EPP caused localized edema of the ear within 15 min and reached a maximum thickness at 60 min after induction. As shown in Figure 3, treatment with PBZ, which is a standard anti-inflammatory drug, markedly reduced ear edema thickness when compared with the DMSO control (*P*<0.05) at all assessment times. PFO significantly decreased ear edema thickness in a dose-dependent manner at all evaluation times, while PFO-SM slightly inhibited ear edema only at the first 15 min after EPP application (*P*>0.05). The maximum degrees of inhibition of ear edema by PBZ, PFO at 0.1 and 1 mL/ear, and PFO-SM were evident at 15 min at values of 50.9, 59.3, 65.7 and 25.9%, respectively. Indeed, PFO treatment at a dose of 1 mL/ear seemed to present more potent anti-inflammatory activities than PBZ treatment at 15, 30 and 60 min (*P*>0.05). These findings imply that PFO could exert anti-inflammatory activity, resulting in the inhibition of ear edema in rats.

#### Carrageenan-induced hind paw edema in rats

**Figure 4:**
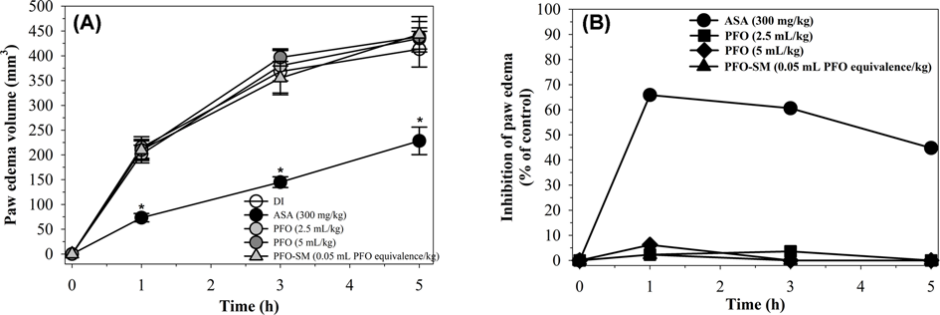
Anti-inflammatory effects of PFO, PFO-SM and ASA on carrageenan-induced hind paw edema in rats Male rats (*n*=6, each) were fed with DI, PFO (2.5 and 5 mL/kg), PFO-SM (0.05 mL PFO equivalence/kg) and ASA (300 mg/kg), then induced by intradermal injection of carrageenan (1%, 50 µL/paw) into the right hind paws of rats, and measured the paw edema volume using a plethysmometer. Statistics was performed by one-way ANOVA test, which *P*<0.05 was considered significance. Data of paw edema volume are expressed as mean ± SD, which *P<0.05 when compared with DI (**A**). Percentage of inhibition of paw edema are shown (**B**).

As shown in Figure 4, injection of carrageenan led to hind paw edema with the maximum paw size occurring at 3 h after induction. Pretreatment of ASA significantly decreased edema volume of the paws when compared with the DI control at all evaluation times at 65.9, 60.6 and 44.8% inhibition of paw edema at 1, 3 and 5 h, respectively after carrageenan injection. However, PFO and PFO-SM did not show any inhibitory effect on carrageenan-induced hind paw edema in rats.

### Anti-ulcer activity of PFO and PFO-SM

**Figure 5:**
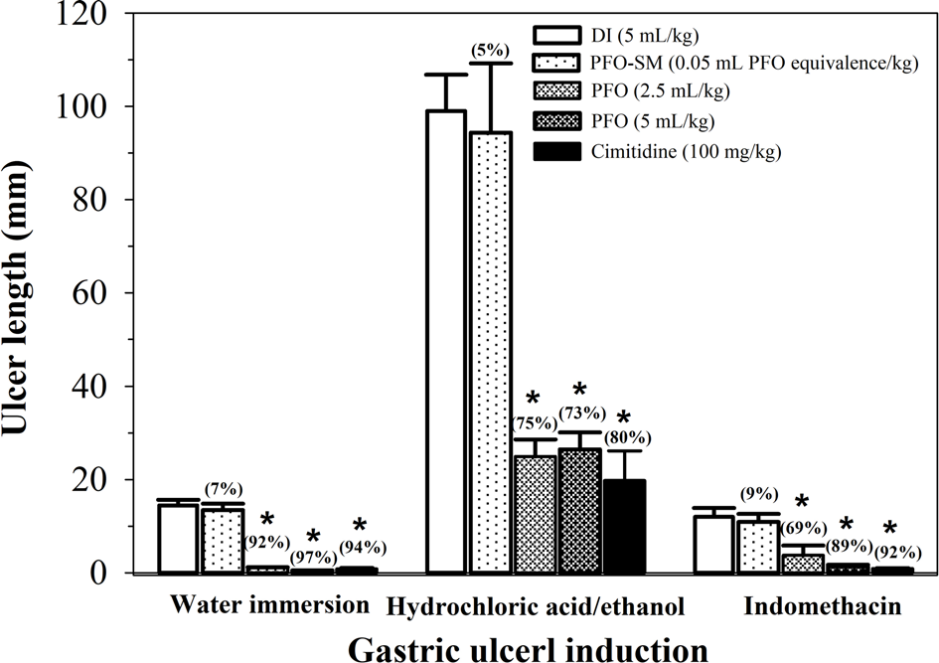
Anti-gastric ulcer effects of PFO, PFO-SM and cimetidine on water immersion, HCl/ethanol and indomethacin-induced gastric ulcer in rats Male rats were induced with gastric ulceration by water immersion, feeding with HCl/ethanol (36.5% in ethanol, 5 mL/kg) or intraperitoneal injection of indomethacin (30 mg/kg). The rats were then treated with DI, PFO (2.5 and 5 mL/kg), PFO-SM (0.05 mL PFO equivalence/kg) and cimetidine (100 mg/kg). The rats were killed by intraperitoneal injection of pentobarbital sodium (40 mg/kg) and measured gastric ulcer length. Statistics was performed by one-way ANOVA test, which *P*<0.05 was considered significant. Data of gastric ulcer length are expressed as mean ± SD. *P<0.05 when compared with DI. Percentage of inhibition of the gastric ulceration are shown in the bracket.

Anti-ulcer activities of PFO and PFO-SM were investigated in cases involving water immersion-induced stress, HCl/ethanol and indomethacin-induced rats, and the results are shown in Figure 5.

#### Water immersion stress-induced gastric ulcer in rats

Apparently, immersion of rats in water for 5 h produced gastric lesions, while pretreatment of the rats with cimetidine as a standard gastroprotective drug significantly decreased the lengths of the lesions at 94.5% inhibition of the gastric ulcer. Surprisingly, PFO significantly reduced gastric ulcer length in a concentration-dependent manner when compared with the DI control group. PFO at 2.5 and 5 mL/kg exhibited 92.4 and 96.6% inhibition of gastric ulcer. Indeed, the gastroprotective effect of PFO was nearly the same as that of cimetidine; however, PFO-SM (0.05 mL PFO eqivalence/kg) resulted in a low degree of anti-ulcer (6.9% inhibition).

#### HCl/ethanol-induced gastric ulcer in rats

As shown in Figure 5, HCl/ethanol remarkably induced gastric lesions in rats. Pretreatments with cimetidine and PFO (2.5 and 5 mL/kg) markedly decreased gastric lesions when compared with the DI control (*P*<0.05), resulting in 80.0, 74.8 and 73.3% inhibition, respectively. Similarly, PFO-SM (0.05 mL PFO equivalence/kg) showed slight protective effects against gastric ulcer (4.7% inhibition).

#### Indomethacin-induced gastric ulcer in rats

As shown in Figure 5, injection of indomethacin caused gastric ulcer in rats. Cimetidine significantly decreased gastric lesions at 92.5% inhibition. Pretreatment with PFO (2.5 and 5 mL/kg) effectively prevented gastric lesions (*P*<0.05) in a dose-dependent manner (68.8 and 88.9% inhibition, respectively); nonetheless, PFO-SM (0.05 mL PFO equivalence/kg) slightly exhibited anti-ulcer (9% inhibition) when compared with the DI control group.

## Discussion

The present study has demonstrated the chemical constituents and biological activities of Thai *Perilla frutescence* fruit oil in animals. HPLC analysis of PFO revealed two predominant essential PUFAs including ALA (ω3-PUFA) and LA (ω6-PUFA). In accordance, ALA and LA exist not only in Thai PFO, but also in Korean PFO [11,28]. It has been well documented that consumption of oil with a high ratio of ω6-PUFA:ω3-PUFA is associated with a high risk of chronic inflammatory diseases [9]. In the present study, the Thai PFO had a low ratio (0.38) of ω6-PUFA:ω3-PUFA, while other PFO had a ratio of 0.25 [32,33]. Remarkably, this ratio difference may possibly be due to a variety of cultivars and environmental factors that are present during the cultivation process, such as temperature, light and existing ground minerals. In addition, we have previously demonstrated that Thai PFO was abundant with vitamin E including β-tocopherol (49.50 mg/kg), α-tocopherol (25.05 mg/kg) and γ-tocotrienol (43.65 mg/kg) [18]. With regard to consumer safety, we found that Thai PFO did not cause acute toxicity in mice in which the LD_50_ value was greater than 10 mL/kg. This result is in agreement with previous study found that perilla oil at doses of up to 50 mL/kg did not induce toxicity in mice [34]. Taken together, these findings imply that Thai PFO, which rich in ALA, LA and vitamin E (mainly β-tocopherol, α-tocopherol and γ-tocotrienol), is beneficial and safe for nutritious and pharmacological purposes.

Pain is an unpleasant sensory and emotional experience; however, it is an important defense mechanism that serves to warn the body about the presence of actual injury or disease. It can also persist and can consequently affect one’s daily activities as well as individual’s quality of life. Consequently, analgesics are often prescribed to prevent or minimize pain. An acetic acid-induced writhing model is commonly used to evaluate analgesic activity in animals, as writhing is a responsive indicator of pain that is induced by certain irritants such as acetic acid. Normally, acetic acid can damage tissues and induce the release of inflammatory mediators such as histamine, bradykinin and PGs. These mediators essentially stimulate nociceptors (known as pain-sensing nerve cells), which will further transmit signals to the spinal cord and brain in response to pain. ASA or aspirin, which is a well-known analgesic and NSAID drug, can irreversibly and non-selectively inhibit the activity of cyclooxygenases (COXs) that catalyze the conversion of arachidonic acid (AA) into powerful inflammatory mediators, especially PGs [35]. Herein, the administration of ASA (150 mg/kg) potently inhibited writhing by up to 80%, while PFO and PFO-SM weakly inhibited writhing suggesting that the bioactive compounds present in PFO and PFO-SM could inhibit the production of inflammatory mediators and lead to an analgesic effect. Presumably, ALA, LA and vitamin E may modulate the production of inflammatory mediators and respond to the analgesic activity of PFO and PFO-SM. In cells, the conversions of ALA into eicosapentanoic acid (EPA) or LA to AA, are catalyzed by serial Δ-6-desaturase, elongase and Δ-5-desaturase enzymes, of which the latter products are known to be further converted into PGs by COX [36,37]. However, the 3-series PGs which derived from EPA, exert fewer pro-inflammatory or anti-inflammatory properties than the 2-series PGs which derived from AA [38]. Indeed, EPA could act as a competitive inhibitor of AA to inhibit the activity of COX leading to a decrease in PGE_2_ production, but also results in an increase in PGE_3_ synthesis [39]. A previous study has claimed that the intake of fish oil rich in ALA, EPA and docosahexaenoic acid (DHA) could prevent colon cancer in rats through inhibition of the synthesis of pro-inflammatory mediators, especially PGE_2_ [40]. In addition to essential fatty acids, vitamin E also displayed an anti-inflammatory property through the inhibition of PGE_2_ [41]. Apparently, the findings indicate that PFO and PFO-SM display analgesic effects in acetic acid-induced writhing in mice.

In addition to the analgesic activity, we also evaluated the anti-inflammatory activity of PFO and PFO-SM using EPP-induced ear edema and carrageenan-induced hind paw edema in rats. EPP and carrageenan are widely used to induce acute inflammation in rats. Topical applications of EPP to the irritated skin of the ears of rats further induced the release of various kinds of inflammatory mediators including histamine, bradykinin and PGs. All of these mediators increased vascular permeability, plasma leakage and incidences of edema. They also promoted the infiltration of leukocytes to the site of the injury, especially monocytes and neutrophils, which can subsequently generate pro-inflammatory cytokines and reactive oxygen species (ROS), leading to the amplification of an inflammation response. In the present research work, both PBZ and PFO significantly suppressed EPP-induced ear edema. PBZ is an NSAID that is known to inactivate COX and inhibit the synthesis of PGs. It is likely that PFO, containing ALA and vitamin E, may effectively inhibit ear edema by reducing the levels of PGs and pro-inflammatory cytokines, as well as by scavenging ROS. This hypothesis is supported by the findings of previous reports which found that perilla oil suppressed the production of pro-inflammatory cytokines including the tumor necrosis factor (TNF)-α, interleukin (IL)-1β and IL-6 in the bronchoalveolar lavage fluid of ovalbumin-challenged mice [15]. One study reported that a high intake of ALA reduced the production of pro-inflammatory cytokines including TNF-α, IL-1β and IL-6 in peripheral blood mononuclear cells of hypercholesterolemic subjects [42]. Additionally, several studies have found that vitamin E not only exerted strong anti-oxidant activity, but also displayed anti-inflammatory properties through the inhibition of TNF-α and IL-6 [43–45]. As mentioned above, data are in good agreement with regard to the anti-inflammatory effects of chemically detected PFO that have been identified in the present study. In comparison with PFO, PFO-SM, that contained 100-fold less the amount of PFO, only slightly inhibited ear edema in the first 15 min after the topical application of EPP. Furthermore, we also evaluated the effects of PFO on carrageenan-induced hind paw edema in rats. Carrageenan is a mucopolysaccharide that is extracted from *Chondrus crispus*, which can then induce the release of histamine, bradykinin and PGs, resulting in increases in vascular permeability and inflammatory cell infiltration. In our experiment, pretreatment with aspirin significantly inhibited carrageenan-induced hind paw edema in rats. Unfortunately, both PFO and PFO-SM had no effect on paw edema in rats. It is likely that PFO would need time to be absorbed and metabolized before being transported to various types of cells for a range of purposes. The findings suggest that PFO displays anti-inflammatory activities in EPP-induced ear edema but had no effect on carrageenan-induced hind paw edema in rats.

Furthermore, we investigated the gastroprotective effects of PFO and PFO-SM on water immersion stress-induced gastric ulcers, HCl/ethanol-induced gastric ulcers and indomethacin-induced gastric ulcers in rats. In the present study, we used different kinds of ulcer inducers to promote gastric injuries including those that were induced by stress, alcohol and NSAIDs like indomethacin [5–7]. Alcohol and indomethacin are known to destroy gastric mucosa, while stress promotes the secretion of stomach acid, which can result in tissue injuries [4,6]. These gastric injuries involve inflammation and oxidative stress through the generation of pro-inflammatory cytokines as well as ROS. The current study has shown that cimetidine and PFO effectively protected against gastric ulcers induced by water immersion stress, HCl/ethanol and indomethacin in rats. As a positive drug, cimetidine is a well-known gastric acid reducer that has been used to treat gastric ulcers. In our findings, active compounds detected in PFO exert anti-inflammatory and anti-oxidant activities, leading to the prevention of gastric ulcers. Indeed, previous evidence supports the claim that the anti-inflammatory activity of PUFAs, like ALA and LA, are directly associated with the number of double bonds that are present in their structures [46]. ALA, LA and DHA suppressed both IL-8 mRNA and protein expression in *H. pylori*-infected gastric epithelial cells, whereas DHA displayed the greatest degree of suppression of IL-8 production followed by ALA and LA, respectively [46]. It has been reported that perilla oil as well as ALA displayed protective effects against hydrogen peroxide-induced neuronal oxidative stress and apoptosis in human neuroblastoma (SH-SY5Y) cells [47]. Vitamin E also protected against gastric mucosa injury by scavenging ROS and lowering lipid peroxidation in ethanol-induced gastric ulcers in rats [48,49]. Indeed, PFO effectively ameliorated gastric ulcers in rats induced by water immersion stress, HCl/ethanol and indomethacin through anti-inflammatory and anti-oxidant activities.

Importantly, it has been reported that a high intake of ALA is associated with lower production of inflammatory mediators and cytokines. These inflammatory mediators and cytokines are involved in the amplification of inflammatory responses [42,50]. ALA, which is a major PUFA in PFO, has been known to be enzymatically converted into 3ω-PUFA, such as EPA and DHA, in the body. In addition, these enzymes share their functions in metabolizing LA to AA, which will then be further converted into inflammatory mediators (eicosanoids). Thus, a high intake of ALA could reduce the production of inflammatory mediators derived from AA and consequently inhibit inflammation. Interestingly, DHA was found to inactivate nucleotide-binding domain and leucine-rich repeat containing protein-3 (NLRP3) inflammasome which is assembled in response to infection or danger signals and subsequently activates the maturation and release of many pro-inflammatory cytokines [51,52]. With regard to the anti-inflammatory property of PFO, ALA and its metabolites (EPA and DHA) transduce a signal through a G-protein-coupled receptor 120 and then activate their downstream scaffold protein β-arrestin-2. β-arrestin-2 subsequently binds to NLRP3 inflammasomes, resulting in inactivation of NLRP3 inflammasome, caspase-1 and inflammatory cytokines production. Furthermore, 3ω-PUFA has been reported to suppress the nuclear translocation of nuclear factor-κB, which is a priming step of inflammasome activation and leads to the inhibition of pro-inflammatory cytokines [52].

## Conclusions

The present study demonstrates that Thai PFO, containing ALA and LA, did not cause acute toxicity in mice. Pretreatment with PFO and PFO-SM inhibited acetic acid-induced writhing in mice, in which the PFO-SM was more potently analgesic than the PFO. Importantly, PFO effectively suppressed EPP-induced ear edema and prevented gastric ulcers induced by water immersion stress, HCl/ethanol and indomethacin in rats. Taken together, the findings from this present study reveal the analgesic effect of PFO and PFO-SM, as well as the anti-inflammatory and anti-ulcer activities of PFO. Furthermore, PFO and PFO-SM should be urgently investigated for their potent analgesic, anti-inflammatory and anti-ulcer effects in humans.

## Data Availability

The authors confirm that the data supporting the findings of the present study are available within the article.

## Abbreviations

18-crown-6: 1,4,7,10,13,16-hexaoxacyclooctadecane
AA: arachidonic acid
ALA: α-linolenic acid
ASA: acetylsalicylic acid
Br-MMC: 4-bromomethyl-7-methoxycoumarin
COX: cyclooxygenase
DHA: docosahexaenoic acid
DI: deionized water
DMSO: dimethylsulfoxide
EPA: eicosapentanoic acid
EPP: ethyl phenylpropiolate
HCl: hydrochloric acid
HPLC: high-performance liquid chromatography
HPLC/FLD: high-performance liquid chromatography/fluorescence detection
IL: interleukin
LA: linoleic acid
LD_50_: median lethal dose
NLRP3: nucleotide-binding domain and leucine-rich repeat containing protein-3
NSAID: non-steroidal anti-inflammatory drug
PBZ: phenylbutazone
PFO: Perilla fruit oil
PFO-SM: PFO-supplemented soybean milk
PG: prostaglandin
PUFA: polyunsaturated fatty acid
ROS: reactive oxygen species
SM: soybean milk
TNF: tumor necrosis factor
T_R_: retention time
